# Phenomenology and anatomy of abnormal behaviours in primary progressive aphasia

**DOI:** 10.1016/j.jns.2010.03.012

**Published:** 2010-06-15

**Authors:** Jonathan D. Rohrer, Jason D. Warren

**Affiliations:** Dementia Research Centre, Department of Neurodegenerative Disease, UCL Institute of Neurology, University College London, Queen Square, London, WC1N 3BG, UK

**Keywords:** Primary progressive aphasia, Frontotemporal lobar degeneration, Frontotemporal dementia

## Abstract

Primary progressive aphasia (PPA) is a group of disorders with progressive language impairment. Abnormal behaviour may develop in PPA as the disease evolves, but the clinical features and brain basis of behavioural change in PPA have not been fully defined. 33 PPA patients (9 semantic dementia, SD, 14 progressive nonfluent aphasia, PNFA, 7 logopenic/phonological aphasia, LPA and 3 patients with a PPA syndrome in association with progranulin mutations, *GRN*-PPA) were assessed using the Neuropsychiatric Inventory to record behavioural changes, as well as volumetric MR imaging. The most common abnormal behaviours in SD were irritability, disinhibition, depression and abnormal appetite, in PNFA apathy, agitation and depression, in LPA anxiety, irritability, agitation and apathy, and in *GRN*-PPA apathy and irritability. Voxel-based morphometry analysis revealed greater atrophy of right lateral orbitofrontal cortex (OFC) in PPA patients with anxiety, apathy, irritability/lability and abnormal appetite/eating disorders, and greater atrophy of left OFC in those with disinhibition. Areas involved beyond OFC included right dorsolateral prefrontal cortex (apathy), right cingulate (irritability/lability) and left anterior superior and medial temporal lobe (disinhibition). Behavioural abnormalities may be clinically significant in PPA, and these abnormalities are underpinned by atrophy of overlapping frontotemporal networks centred on OFC.

## 1 Introduction

Primary progressive aphasia (PPA) is a group of neurodegenerative disorders with language impairment as the dominant feature [1–3] and is generally considered to fall within the frontotemporal lobar degeneration (FTLD) spectrum of disorders [4]. Distinct profiles of language impairment define the three canonical subtypes of PPA: anomia and comprehension deficits due to semantic knowledge loss, semantic dementia (SD); apraxia of speech and agrammatism, progressive nonfluent aphasia (PNFA); and word-finding difficulty with impaired repetition and comprehension of sentences, logopenic/phonological aphasia (LPA). These are usually sporadic syndromes but PPA may occasionally be associated with mutations in the progranulin (*GRN*) gene (*GRN-*PPA). PPA is most commonly associated with the non-Alzheimer FTLD pathologies, i.e. tau-positive or TDP-43 positive inclusions, but LPA in particular can be associated with Alzheimer's disease pathology i.e. amyloid plaques and tau-positive neurofibrillary tangles [5]. The PPA syndromes have corresponding patterns of regional atrophy affecting dominant hemisphere language networks: anteroinferior temporal lobe involvement in SD; inferior frontal, insula and superior temporal lobe involvement in PNFA; posterior temporal and inferior parietal lobe involvement in LPA; and more widespread frontal, temporal and parietal involvement in *GRN*-PPA. Disease progression is associated with more widespread left hemisphere atrophy and increasing involvement of the right (nondominant) hemisphere [6,7]. Clinically, evolution is associated with increasing prominence of cognitive deficits beyond the domain of language and the development of behavioural abnormalities [8,9].

In contrast to behavioural variant frontotemporal dementia (bvFTD) there have been relatively few studies of the phenomenology and brain basis of behavioural abnormalities in PPA [8–12]. Here we describe clinical behavioural profiles in each of the subtypes of PPA and assess the neuroanatomical correlates of behavioural change in PPA using voxel-based morphometry. Accumulating neuroanatomical evidence suggests that complex behaviours in neurodegenerative disease are mediated by frontotemporal networks, in particular, orbitofrontal cortex (OFC) and limbic structures with a right hemisphere emphasis [13–20]. Informed by this previous work, our core neuroanatomical hypothesis here was that behavioural disturbances in PPA syndromes are associated with atrophy of OFC and its functional connections.

## 2 Methods

Thirty three consecutive patients with a diagnosis of PPA (9 with SD, 14 with PNFA, 7 with LPA and 3 with *GRN*-PPA) according to current consensus clinical and neuropsychological criteria [2–4,21–23] participated. Cases were ascertained via a larger longitudinal neuropsychological and neuroimaging study of PPA, findings from which have been previously reported in part [24]. All patients were administered the Neuropsychiatric Inventory (NPI, [25]), a questionnaire examining the presence and severity of the following abnormal behaviours: delusions, hallucinations, agitation/aggression, depression/dysphoria, anxiety, elation/euphoria, apathy/indifference, disinhibition, irritability/lability, aberrant motor behaviour, abnormal sleep and abnormal appetite/eating behaviours. Apart from one patient with *GRN*-PPA who had a cardiac pacemaker all patients also had volumetric brain MRI. Demographic data in each of the subgroups are presented in Table 1. Research ethics approval for this study was obtained from the National Hospital for Neurology and Neurosurgery and University College London Hospitals Research Ethics Committees.

Voxel-based morphometry (VBM) was performed on the patients' brain MR images using SPM5 software (http://www.fil.ion.ucl.ac.uk/spm) and the DARTEL toolbox with default settings for all parameters [24,26,27]. The images were smoothed using a 6 mm full-width at half-maximum (FWHM) Gaussian kernel and linear regression models used to examine differences in GM intensity correlating with the presence of frequently abnormal behaviours (behaviour exhibited by >25% of the PPA cohort, as indexed by the NPI). For each behaviour, subjects were classified according to whether they did or did not exhibit that behaviour and the contrast of interest was the difference between these two groups. Voxel intensity, *V*, was modelled as a function of group, and subject age and total intracranial volume were included as nuisance covariates. *V* = *β*_1_ abnormal behaviour + *β*_2_ no abnormal behaviour +*β*_3_ age + *β*_4_ TIV + *µ* + *ε* (where *µ* is a constant, and *ε* is error). Maps showing statistically significant differences between the groups were generated uncorrected at *p* < 0.001 significance level. Statistical parametric maps were displayed as overlays on a study-specific template, created by warping all native space whole-brain images to the final DARTEL template and calculating the average of the warped brain images.

## 3 Results

Abnormal behaviours exhibited by patients across the PPA cohort are summarised in Table 2: the mean NPI score for patients exhibiting the behaviour (an index of behaviour salience, where each score is the mean product of individual scores [behaviour severity × behaviour frequency]) and the proportion of patients exhibiting each behaviour (an index of behaviour prevalence in that patient group) are shown. The most prevalent and salient behaviours across the PPA cohort were agitation/aggression, depression, anxiety, apathy, disinhibition, irritability/lability, and abnormal appetite/eating disorders (Table 2). Total NPI score varied between 0 (in 5 patients) and 45. There was no relationship between total score and either duration of disease or a measure of disease severity (MMSE score), either in the PPA cohort as a whole or in any of the subgroups.

All patients with SD and a majority of patients in each of the PNFA, LPA and *GRN*-PPA subgroups exhibited at least one abnormal behaviour: the overall prevalence and salience of abnormal behaviours was similar between PPA subgroups as was the overall amount of caregiver distress created by the behaviours (Table 2). Most behaviours were exhibited by all PPA subgroups and none was wholly specific for a particular subgroup. However, different profiles of behavioural change were observed between subgroups. The most prevalent behaviours in each subgroup (defined arbitrarily as behaviours exhibited by at least half the patients in that subgroup), were: in SD (in rank order), depression, irritability/lability, disinhibition, abnormal appetite/eating disorders and anxiety; in PNFA, apathy, depression and agitation/aggression; in LPA, irritability/lability, anxiety, apathy and agitation/aggression; and in *GRN*-PPA, apathy and irritability/lability (Table 2).

**Fig.1:**
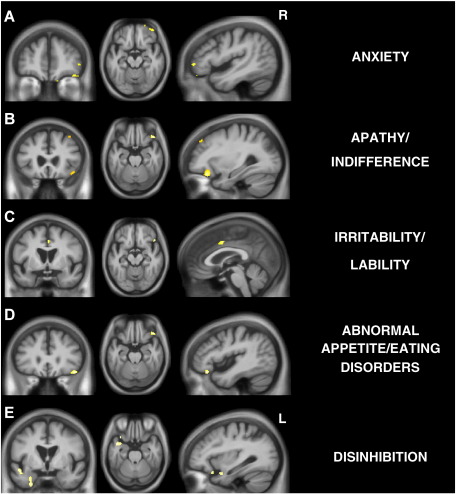
VBM analyses on grey matter regions in contrasts based on presence versus absence of abnormal behaviours as shown. Statistical parametric maps (SPMs) have been thresholded at *p* < 0.001 (uncorrected) and rendered on a study-specific average group T1-weighted MRI template image in DARTEL space. In coronal and axial sections, the right hemisphere (R) is shown on the right side of the image. Left (L) and right (R) markers are shown for the sagittal sections.

As the overall most prevalent abnormal behaviours were exhibited by all PPA subgroups, the subgroups were merged in the VBM analysis, in order to assess regional atrophy that correlated with the emergence of the behaviour for the PPA cohort as a whole. No VBM correlates were identified for the presence of depression or agitation/aggression (*p* < 0.001 uncorrected). However partly overlapping VBM correlates were identified for other frequently abnormal behaviours (*p* < 0.001 uncorrected) (Fig. 1) in accord with our a priori anatomical hypotheses [13–20]. The presence of anxiety, apathy, irritability/lability, and abnormal appetite/eating disorders all correlated with reduced grey matter intensity in right lateral OFC (Fig. 1A–D), while the presence of disinhibition correlated with reduced grey matter in left lateral OFC (Fig. 1E). Additional areas of grey matter loss correlating specifically with the presence of particular behaviours were identified: the presence of apathy correlated with reduced grey matter intensity in right dorsolateral prefrontal cortex (Fig. 1B); the presence of irritability/lability correlated with reduced grey matter intensity in right anterior cingulate (Fig. 1C); and the presence of disinhibition correlated with reduced grey matter intensity in left anterior superior temporal gyrus and entorhinal cortex (Fig. 1E).

## 4 Discussion

This study demonstrates that abnormal behaviour can develop in any of the canonical subtypes of PPA. While particular PPA subtypes did not show an overall predilection to develop behavioural abnormalities, partly differentiable profiles of behavioural impairment were associated with different subtypes. In previous work, abnormal eating patterns and disinhibition have also been associated, as here, with SD [10,12]. However, in contrast to earlier work using the NPI [12] we did not find a substantial overall increase in behavioural dysfunction in SD compared with the other groups. This may be due in part to the fact that our sample only contained patients with SD and left-sided predominant temporal lobe atrophy (no patients with SD and right-sided predominant temporal lobe atrophy presented during the period of this study): the previous study by Rosen et al. [12] may have included patients with greater right temporal lobe involvement and more prominent behavioural symptoms [9]. The greater variability in disease duration in the earlier sample may also have contributed, though we did not find a clear relation between behavioural disturbance and disease duration in the SD sample here. More speculatively, other factors such as cultural norms (Rosen et al., 2006 [12] studied a US patient cohort) may also have influenced reporting of particular behaviours, such as changes in eating behaviour. The findings in the PNFA and LPA groups in this study also overlapped with those in the previous study of PPA [12]: in particular apathy scored highly for each group in both studies. However in contrast to the study of Rosen et al. [12], anxiety and irritability also scored highly in our LPA group.

Previous studies addressing the neuroanatomical correlates of behavioural impairment in dementia have implicated a predominantly right-sided network of frontal (particularly OFC), cingulate and striatal areas in the pathogenesis of apathy, disinhibition and abnormal appetite [13–19]. The present data corroborate these previous findings, and underline the critical role of right OFC damage in the production of a range of abnormal behaviours in PPA. It has been proposed that OFC is involved in processing stimulus–reward associations: neuronal loss in this area leads to impaired ability to make such associations, with resulting abnormal behaviour [20]. It has been further proposed that lateral OFC may be involved in organising behaviour toward a goal, while medial OFC evaluates the outcome [28], suggesting that lateral OFC may play a generic role in the regulation of different kinds of behavioural output. Damage involving lateral OFC is therefore predicted to be associated (as here) with the emergence of a range of disorganised or context-inappropriate behaviours. The additional specific correlates of abnormal behaviours identified here may signify brain areas with more specific roles in the pathogenesis of particular abnormal behaviours, consistent with previous clinical studies and with emerging concepts of the cerebral organisation of these behaviours: dorsolateral prefrontal cortex damage has previously been linked with apathy [17], anterior cingulate dysfunction has been associated with emotional lability [29], and entorhinal cortex participates in cerebral networks that mediate adaptive avoidance behaviours [30], These partly differentiated neuroanatomical correlates may also help explain or predict the relative prominence of particular behaviours in different PPA subgroups (for example, apathy would be more likely with more prominent dorsal prefrontal dysfunction in PNFA and LPA, disinhibition with mesial temporal neocortical dysfunction in SD).

The issue of cerebral lateralisation is more problematic: neuroanatomical correlates of abnormal behaviour, in the present and in previous studies, are predominantly located in the right hemisphere, however disinhibition here correlated with damage in a left-sided frontotemporal network. Disinhibition might result from impaired ability to make affect-incongruent responses, a role attributed to left OFC in normal subjects [31]. Clinically, the present findings suggest that the primacy of right hemisphere damage in the pathogenesis of abnormal behaviour is relative rather than absolute. It is noteworthy that those abnormal behaviours correlating with right hemisphere atrophy in the present study (anxiety, apathy, irritability, and appetite) might broadly result from deranged processing of internally generated (e.g. affective) cues, while the behaviour correlating with left hemisphere damage (disinhibition) results from deranged processing of external (environmental) cues. This suggests a possible pathophysiological basis for the differential lateralisation we observed that is broadly consistent with other lines of evidence in affective neuroscience [32].

The PPA syndromes are likely to overlap anatomically and histopathologically with bvFTD, in which behavioural disturbances are an early and defining feature [4,9]. An anterior-cingulate fronto-insular network with projection zones including OFC has been implicated as a critical substrate in bvFTD [33]. In light of the present findings in PPA, the relative preponderance of language versus behavioural phenomenology in the various syndromic variants of FTLD might reflect differential involvement of common cerebral networks. This issue should be explored in future longitudinal studies of behavioural impairment in PPA, including techniques such as diffusion tractography and functional MRI that can capture structure:function relations in the distributed neural networks that mediate complex behaviours. No less important than the application of new imaging techniques will be the development of more sensitive behavioural metrics: the NPI, though widely used and validated, provides relatively limited scope for detailed analysis of particular behaviours, and fresh insights may depend on the application of new instruments tailored to FTLD and other dementia populations.

## Figures and Tables

**Table 1:** Demographic data of patients.

Mean (standard deviation)	SD	PNFA	LPA	GRN-PPA
Number of subjects	9	14	7	3
%Male	33.3	71.4	57.1	66.6
Age (years)	62.3 (9.0)	71.8 (6.8)	65.1 (6.4)	61.6 (9.1)
Duration (years)	5.3 (1.2)	5.3 (2.1)	4.4 (1.0)	3.9 (0.3)
MMSE (/30)	22.7 (5.2)	24.4 (5.6)	13.8 (5.7)	17.0 (2.6)

**Table 2:** NPI mean (standard deviation, StDev) scores and percentage of patients exhibiting abnormal behaviour in all PPA patients and in the PPA subgroups. Behaviours exhibited by ≥50% of patients in each subgroup are indicated in bold. The NPI score is based on use of discrete scales: for each behaviour, the score (individual behaviours/12, total/144) shown is the mean product of individual scores on scales of severity [1, mild – 3, severe] × frequency [1, occasionally – 4, very frequently]; for severity of caregiver distress [total scores/60 only shown], 0, no distress – 5, extremely distressing.

	ALL		SD		PNFA		LPA		GRN-PPA	
	Mean (StDev)	%	Mean (StDev)	%	Mean (StDev)	%	Mean (StDev)	%	Mean (StDev)	%
Delusions	0.4 (1.3)	9	0.4 (1.3)	11	0.4 (1.6)	7	0.4 (1.1)	14	0.0 (0.0)	0
Hallucinations	0.1 (0.4)	6	0.2 (0.7)	11	0.0 (0.0)	0	0.1 (0.4)	14	0.0 (0.0)	0
Agitation/aggression	0.9 (1.4)	50	0.7 (1.0)	44	**0.9 (1.2)**	**50**	**1.4 (2.1)**	**57**	0.3 (0.6)	33
Depression/dysphoria	1.3 (1.8)	56	**1.1 (0.9)**	**78**	**1.6 (2.2)**	**57**	0.4 (0.8)	29	2.0 (3.5)	33
Anxiety	1.2 (1.8)	50	**0.8 (0.8)**	**56**	0.8 (1.6)	36	**2.6 (2.6)**	**71**	0.7 (1.2)	33
Elation/euphoria	0.6 (1.6)	19	0.8 (2.0)	22	0.5 (1.6)	14	0.1 (0.4)	14	1.3 (2.3)	33
Apathy/indifference	1.7 (2.4)	56	0.7 (1.1)	33	**2.1 (2.9)**	**64**	**2.0 (2.8)**	**57**	**1.7 (1.5)**	**67**
Disinhibition	1.3 (2.5)	38	**2.0 (2.9)**	**67**	0.7 (2.4)	14	1.7 (2.4)	43	0.7 (1.2)	33
Irritability/lability	1.4 (1.9)	56	**1.2 (1.4)**	**78**	0.9 (2.1)	29	**2.4 (2.3)**	**71**	**1.3 (1.2)**	**67**
Aberrant motor behaviour	0.7 (1.6)	22	0.3 (0.7)	22	0.5 (1.2)	21	1.7 (2.9)	29	0.0 (0.0)	0
Abnormal sleep	0.8 (1.6)	25	1.1 (1.5)	44	0.8 (1.6)	21	0.9 (2.3)	14	0.0 (0.0)	0
Abnormal appetite/eating disorders	2.0 (3.1)	50	**1.7 (1.6)**	**67**	2.6 (4.3)	43	1.4 (2.3)	43	1.3 (2.3)	33
*Total*	*12.2 (12.4)*	*88*	*11.0 (7.4)*	*100*	*11.9 (14.8)*	*79*	*15.3 (15.3)*	*86*	*9.3 (8.1)*	*67*
*Caregiver distress total*	*9.2 (6.0)*	*88*	*9.4 (6.1)*	*100*	*7.4 (6.0)*	*79*	*9.6 (6.8)*	*86*	*5.3 (4.60*	*67*
